# Potential Role of Meiosis Proteins in Melanoma Chromosomal Instability

**DOI:** 10.1155/2013/190109

**Published:** 2013-06-12

**Authors:** Scott F. Lindsey, Diana M. Byrnes, Mark S. Eller, Ashley M. Rosa, Nitika Dabas, Julia Escandon, James M. Grichnik

**Affiliations:** ^1^Department of Dermatology and Cutaneous Surgery, University of Miami Miller School of Medicine, Miami, FL 33136, USA; ^2^Anna Fund Melanoma Program Sylvester Comprehensive Cancer Center, University of Miami Miller School of Medicine, Miami, FL 33136, USA; ^3^Frankel Family Division of Melanocytic Tumors, Department of Dermatology and Cutaneous Surgery, University of Miami Miller School of Medicine, Miami, FL 33136, USA; ^4^Interdisciplinary Stem Cell Institute, University of Miami Miller School of Medicine, University of Miami, FL 33136, USA

## Abstract

Melanomas demonstrate chromosomal instability (CIN). In fact, CIN can be used to differentiate melanoma from benign nevi. The exact molecular mechanisms that drive CIN in melanoma have yet to be fully elucidated. Cancer/testis antigens are a unique group of germ cell proteins that are found to be primarily expressed in melanoma as compared to benign nevi. The abnormal expression of these germ cell proteins, normally expected only in the testis and ovaries, in somatic cells may lead to interference with normal cellular pathways. Germ cell proteins that may be particularly critical in CIN are meiosis proteins. Here, we review pathways unique to meiosis with a focus on how the aberrant expression of meiosis proteins in normal mitotic cells “meiomitosis” could impact chromosomal instability in melanoma and other cancers.

## 1. Introduction

Melanomas exhibit chromosomal instability (CIN). In fact, CIN is one of the most useful molecular markers to differentiate melanomas from benign nevi [1, 2]. Bastian et al. found that 96.2% of melanomas demonstrated chromosomal aberrations while only 13.0% of benign nevi showed these same abnormalities, of which all were Spitz nevi with stable 11p duplications [1]. Melanomas generally exhibit an increased number of overall chromosomes with frequent large translocations [1]. 

The fact that melanomas have such unstable genomes comes as no surprise, as genomic instability is widely regarded as the hallmark of cancer [3–7]. While genomic instability decreases the viability of most cells, it may also permit a subpopulation of cells to acquire genetic changes that lead them to escape normal growth control mechanisms. In addition, genomic instability allows established cancers to evolve and evade immunologic and pharmacologic destruction [8]. The extent to which different mechanisms play a role in genomic instability is controversial [4]; however three pathways are generally most accepted. These are defective DNA repair, telomere crisis, and mitotic spindle malfunction [3, 9–12]. An underappreciated but potentially important research area is the abnormal expression of germ cell proteins.

**Figure 1:**
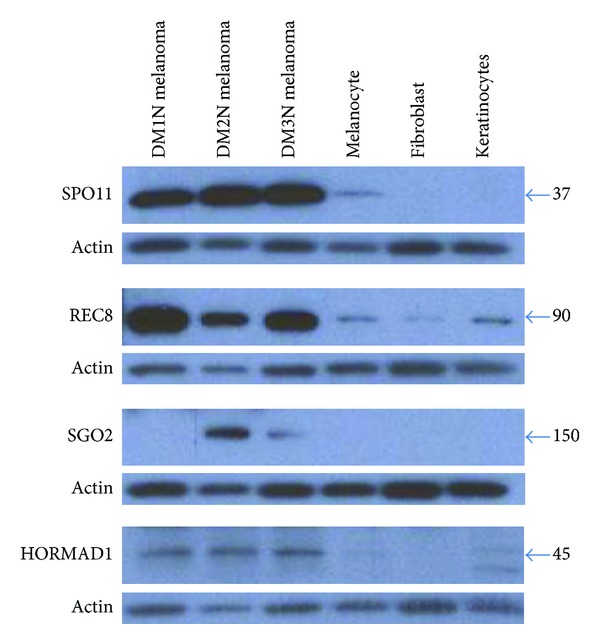
Analysis of meiosis proteins by western blot analysis. Western blot analysis was used to measure the expression of SPO11 (Abcam, ab81695), REC8 (Sigma, HPA031729), SGO2 (Sigma, HPA035163), and HORMAD1 (Abcam, ab57883) in melanoma lines compared to nontransformed melanocytes, fibroblasts, and keratinocytes. The cytoplasmic *β*-actin for each experiment is shown below its respective blot as a loading control.

**Figure 2:**
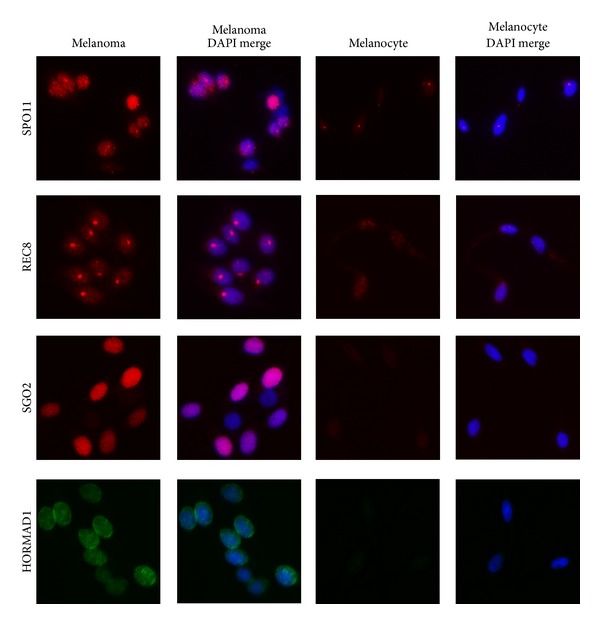
Analysis of meiosis proteins by immunofluorescence. Immunofluorescence was used to analyze the expression of SPO11, REC8, SGO2, and HORMAD1 in the DM2N melanoma line and nontransformed melanocytes using the same antibodies listed in Figure 1. Anti-rabbit secondary antibody was used for SPO11, REC8, and SGO2 and anti-mouse secondary was used for HORMAD1. The staining patterns varied between the different antibodies. SPO11, REC8, and SGO2 stained in a predominately nuclear manner with SPO11 demonstrating a fibular, dotted pattern, REC8 with discrete nuclear dots, and SGO2 with diffuse staining throughout the nucleus. HORMAD1 had a mostly cytoplasmic staining pattern although nuclear staining was also visualized. With all four antibodies, the staining in the melanoma was markedly stronger than the melanocytes.

Expression of germ cell proteins has long been observed in cancer cells [13]. Cancer/testis antigens (CTAs) are a family of germ cell proteins expressed in a multitude of different histological tumor types [13, 14]. These proteins have been noted to have both diagnostic and prognostic value [2]. Studies have shown that expression of specific CTAs in melanoma can be used to predict tumor thickness, the presence of ulceration, and likelihood to undergo metastasis [15]. Melanomas have also been shown to express meiosis specific proteins including SCP1 (homologous chromosome pairing) [16], HORMAD1 (meiotic synapse regulation) [17], SPO11 (double stranded DNA breaks) [18], and REC8 (meiosis cohesion protein) [2]. We have also found three of these meiosis proteins, addition to a fourth not previously described, to be present in melanoma using western blot analysis (Figure 1) and immunofluorescence (Figure 2). Although the potential impact of the expression of meiotic proteins during mitosis in causing CIN has been suggested [17–21] and a role in reductional division in irradiated polyploid cells has been noted [22], thus far there has been no research directly evaluating the potential direct role of meiosis proteins in the creation of CIN. This review is focused on understanding meiosis pathways and explaining how the expression of these pathways during mitosis (“meiomitosis” [19, 20]) may result in chromosomal instability.

## 2. Meiosis General Overview: What Makes Meiosis Different from Mitosis?

Meiosis is a specialized type of cell division. In contrast to mitosis in which a diploid cell (2N) duplicates its DNA once and divides to produce two genetically identical diploid cells (2N), in meiosis a diploid cell (2N) duplicates its DNA once and undergoes two distinct rounds of cell division to produce four genetically unique haploid (1N) cells (Table 1). While mitosis occurs in all somatic cells of the human body, meiosis occurs naturally only in the male testis and female ovary.

The meiosis pathways are thought to have evolved from double-stranded DNA repair pathways [23]. In fact, meiosis still functions as a DNA repair process. This occurs through recombination events, where corresponding sections of DNA on the two homologous chromosomes are exchanged. This ensures that DNA damage is not passed on to progeny [24, 25], in addition to the obvious role of producing genetic diversity to facilitate species evolution [25–27].

Mitosis and meiosis have many similarities (Table 2). The second division of meiosis (meiosis II) has many aspects that are nearly identical to mitosis. The first division, on the other hand, exhibits three major modifications: (i) meiotic pairing to allow recombination, (ii) kinetochore coorientation to allow for homologous chromosome segregation during anaphase I, and (iii) stepwise loss of cohesion to ensure that sister chromatids segregate together in meiosis I [28]. These unique meiosis I pathways are the ones most likely to interfere with normal mitosis and will be the primary focus of this review.

## 3. Meiosis Gene Activation: The Regulatory Switch

The switch to meiosis from mitosis occurs in germ cells when the decision is made to produce gametes. Prior to this switch, germ cells use mitotic divisions as a way of self-renewal to increase their numbers and ensure the cell line does not become depleted [29]. The decision for cells to transition from a mitotic to meiotic cell cycle is complex and differs vastly among organisms. In most simple single-celled organisms that undergo asexual reproduction, this transition occurs as a result of environmental stressors. In yeast, nutrient deprivation triggers the dephosphorylation of the RNA binding protein *Mei2* by *Pat1* Kinase which initiates the switch from mitosis to meiosis [30]. In *Caenorhabditis elegans* germline cells, GLD1 (quaking), GLD2 (Poly(A) polymerase), and GLD3 (Bicaudal C) have been shown to be critical for this switch [31, 32]. In mammals, the decision to transition from mitosis to meiosis is yet more complex and there are numerous regulatory molecules that govern this change. STRA8 is one of these proteins and is known to be imperative for the switch from mitosis to meiosis in both human male and female germ lines [33]. Data from Li et al. support this notion, showing that the spermatocytes in mice with depleted STRA8 failed to enter meiosis [34].

The crucial point in the cell cycle by which the cell must decide to make the mitotic-to-meiotic shift is at the G1/S checkpoint [35]. This is because the cell must undergo meiotic-specific events during S phase to ensure the proper progression of meiosis. The meiotic cohesin protein REC8 (discussed further below) must be incorporated into the newly replicated DNA during the premeiotic S phase so that the kinetochores can orient appropriately to allow for reduction division during meiosis I.

Although meiosis follows mitosis in the production of gametes, it is important to note that in the fertilized egg, mitosis is reestablished after meiosis. Thus the mitosis-meiosis pathways can be switched on and off and are reversible. Dysregulation of this switch causing the collision of the pathways could certainly cause CIN.

## 4. DNA Double Strand Breaks: Rotating the Genes

The formation of crossover events lies at the heart of meiosis, as it allows for genetic recombination between homologous chromosomes and is necessary for the proper alignment of chromosomes during meiosis I. Crossovers are initiated by the creation of double-stranded breaks (DSBs) by the protein SPO11 [36].

SPO11 is a homolog of the archeal type II topoisomerase A subunit [37]. SPO11 creates DSBs using a catalytic tyrosine to attack the phosphodiester backbone of DNA, creating a covalent bond between itself and the 5′ end of the break [37]. Each SPO11 protein associates with only one strand of DNA; thus two SPO11 molecules are needed for each DSB [37]. Once the DSB is formed, SPO11 is removed by polymerase *β* with the help of the MRE11 complex [36, 38] and the 5′ ends of the breaks are excised, leaving stretches of 3′ ssDNA [36]. This single stranded DNA associates with DMC1 and RAD51, two proteins known to be involved in double-stranded DNA repair, to form a filament [37]. This filament then begins the search for a location of homology on the opposite chromosome.

Many of the factors which ensure that the single stranded DNA correctly associates with a corresponding DNA fragment on the homologous chromosome have yet to be elucidated. It is clear that the meiosis specific kinase MEK1 plays a role in ensuring interhomolog binding through its interaction with RAD54 and RAD51 [39, 40]. MEK1 also promotes recombination by suppressing DSB repairs [41]. Upon identification of the appropriate homologue, DMC1 begins the crossover recombination event using one of the 3′ ends to mediate a stable invasion of the homologous chromosome [36]. DNA synthesis then extends the end of the invading strand. By recapture of this strand, a joint molecule is generated that contains a Holiday Junction, which can then be resolved into either a noncrossover or crossover event [36].

HORMAD1 also plays a critical role in synapsis formation through its role in DSB formation [42]. It ensures that adequate number DSBs are formed to allow for successful homology search [42]. Independently, HORMAD1 also promotes the proper formation of the synaptonemal complex, a proteinaceous structure which stabilizes the tetrad and ensures proper homolog pairing [42].

Together these factors cause DNA strand breaks and create a controlled number of crossover events. Deregulation, or simply the presence, of these pathways in otherwise mitotic cells would be expected to cause translocations as well as insertions and deletions. Although there is no data specifically implicating these pathways in cancer, it is important to note that SPO11 [18], DMC1 [22], and HORMAD1 [17] have all been shown to be increased in cancer. Both SPO11 and HORMAD1 have been specifically noted in melanoma [17, 18] and we found them to be overexpressed in melanoma compared to nontransformed cell types using western blot analysis (Figure 1) and immunofluorescence (Figure 2).

## 5. DNA Polymerase **β**: Potential Role Evolving the Genome

At least sixteen DNA polymerases exist in eukaryotes, each with its own unique role in maintaining the integrity of the genome [43]. Specific high fidelity DNA polymerases function in replicating the genome during the S phase of the cell cycle. More error-prone polymerases also exist and have specific functions including DNA repair and cell recombination [43]. While it is imperative that the cells employ methods to preserve the fidelity of DNA replication, error-prone polymerases may play a role in producing variability which could provide a selective advantage [43]. As such, in 1962 Magni and von Borstel observed that cells had a markedly higher rate of mutation during meiosis compared to mitosis [44].

DNA polymerase *β* (POL *β*) is one of the more error prone polymerases found in eukaryotes and is required for base excision repair during DNA replication and repair [45]. It has an error rate of one error per 10,000–100,000 nucleotides incorporated, up to 100x higher than the more accurate polymerases which the cell routinely uses for chromosomal replication [43]. Recently, POL *β* has also been found to have a critical role during meiotic synapsis [45]. POL *β* has been shown to localize to the synaptonemal complex during prophase I [46] where it facilitates the removal of the SPO11 complex from DNA breakage ends, facilitating the creation of ssDNA with which RAD51 and DMC1 associate [47]. Kidane et al. showed that POL *β* knockdown spermatocytes were unable to undergo chromosome synapsis and, instead, underwent apoptosis during prophase I [47].

Deregulated expression of POL *β* has been implicated in genomic instability in cancer [45]. Decreased POL *β* expression has been detected in one fifth of tumors [48] and this decrease has also been demonstrated to promote tumorigenesis [49] presumably due to decreased DNA repair. Conversely, increased levels of POL *β* have been noted in approximately one-third of tumors studied, with one sample showing a 286-fold increase [48]. Luo et al. used mouse embryonic fibroblasts to study the effect of varying levels of POL *β* on genomic instability by looking at three endpoints: DNA strand breaks, chromosomal breakage, and gene mutation [45]. POL *β* null cells showed a marked increase in genomic instability [45] due to their inability to repair DNA. POL *β* overexpressing cells showed a high frequency of mutations, but only after the introduction of DNA damaging agents [45], most likely because POL *β* was used to repair the DNA damage instead of one of its higher fidelity counterparts. 

Servant et al. specifically studied the association between DNA POL *β* and melanoma and found that POL *β* was markedly overexpressed in melanoma tissue when compared to normal skin [50]. Mammalian cells which overexpressed POL *β* had a 1.5–2-fold higher resistance to UV radiation, though the surviving cells had a 2.6–50-fold increase in mutations [50]. POL *β* was able to repair both cyclobutane pyrimidine dimers (CPD) and pyrimidine-pyrimidone (6–4) photoproducts and had the ability to elongate the 3′ strand after dATP addition [50]. This is significant because this period of elongation serves as a potential source of DNA point mutations due to POL *β*'s markedly lower rate of fidelity than other polymerases.

Although POL *β* seems to be expressed at some level ubiquitously in all cells, it clearly plays an integral role in the meiotic pathways. It would be anticipated that cells activating meiosis pathways may exhibit increased POL *β* activity and increased mutations.

## 6. Cohesion: How Not to Let Go of Your Sister

The decision is made to enter the meiotic cycle prior to replicating the DNA. During the replication of DNA for meiosis, the newly duplicated strands are held together by a ring of proteins called cohesins. The mitotic cohesin complex normally includes the structural maintenance (SMC) proteins SMC1 and SMC2, the kleisin protein SCC1/RAD21, and an accessory subunit, SCC3 [51, 52]. In meiosis RAD21 is replaced by REC8 [53].

REC8 serves a number of functions including (1) acting within the synaptonemal complex to drive homologous recombination, (2) kinetochore orientation, and (3) sister chromatid adhesion. 

### 6.1. Synaptonemal Complex/Homologous Recombination

The synaptonemal complex is a unique meiosis specific structure which is critical for chiasma (locations of crossover) formation, homologous chromosome binding, and chromosome segregation [54]. It bridges homologous chromosomes and is composed of three proteins: SCP1, 2, and 3. REC8 is critical in this structure for driving chiasma formation between homologous chromosomes [55, 56]. As the chromosomes condense, REC8 is cleaved on the chromosome arms and the cohesin complex is replaced by the condensin complex. The crossover point remains to ensure proper alignment, tension, and segregation of the homologous chromosomes.

### 6.2. Kinetochore Orientation

REC8 has also been shown to play a role in the monoorientation of sister kinetochores during meiosis I. When the mitotic cohesin protein RAD21 is expressed instead of REC8 in yeast, sister chromatids undergo equational rather than reductional chromosomal segregation during meiosis I [55, 57]. This suggests that the coorientation of kinetochores is lost, allowing sister chromatids to be pulled towards opposite poles of the dividing cell. Studies performed in maize support this theory, demonstrating that, in the absence of REC8, sister chromatids establish bioriented sister kinetochores in meiosis I [58]. While research suggests REC8 is required for monoorientation of sister kinetochores, its overexpression alone does not appear to always lead to chromosomal missegregation during mitosis [55, 57] but instead requires the cooperation of other factors such as monopolins, as reviewed below.

### 6.3. Kinetochore Adhesion

REC8 is retained around the centromere until the start of anaphase II [59] ensuring that sister chromatids do not become prematurely separated. Two of the factors that prevent the cleavage of REC8 at the centromere are SGO1 and SGO2. Although both of these proteins are ubiquitously expressed in mammals, SGO2 is found in higher concentrations in the testis, implying that it may play a more major role in meiosis [60]. Studies have supported this theory, showing that mice with a deleted *Sgo2* gene show no failure to thrive but are infertile, suggesting that this protein is not as critical in mitosis as meiosis [61]. 

The protease separase is required to remove cohesins from chromosomes [62]. REC8 can only be cleaved by separase when the former is hyperphosphorylated [60]. SGO2 protects REC8 from cleavage during meiosis I by ensuring its dephosphorylation at the centromeres. This is accomplished through the recruitment of protein phosphatase 2A (PP2A) to centromere proximal cohesins [28]. PP2A localization to centromeres results in dephosphorylation of centromere proximal REC8, inhibiting its cleavage by separase while allowing REC8 cleavage to occur along chromosome arms.

REC8/Synaptonemal complex proteins could play an important role in chromosomal missegregation and translocations. REC8 expression has been noted in melanoma [2] and we found it to be overexpressed in melanoma using western blot analysis (Figure 1) and immunofluorescence (Figure 2). REC8 has also been noted in irradiated lymphoma cells [22]. Forced expression of REC8 during mitosis leads to chromosome segregation defects [63]. Ishiguro et al. utilized yeast with overproduction of REC8 during mitosis to demonstrate this phenomenon [63]. Compared with the wild type yeast cells, the strains with upregulated REC8 showed bridged nuclei which were not able to divide appropriately [63]. Thus it is possible that high levels of REC8 may play a role in nuclear division abnormalities. 

It has also been postulated that REC8 may play a role in reducing chromosome number in polyploid cancer cells by driving reductional divisions [22]. Interestingly, synaptonemal complex proteins SCP1 and SCP3 have been shown to be upregulated in cancer and are associated with tumor progression and survival [16, 22, 64]. SCP1 is a known cancer/testis antigen [64]. Türeci et al. found that in melanoma, four of the 28 samples tested showed positive synaptonemal complex protein expression [16]. Studies have demonstrated that when inserted into nongerm cells, these proteins are still able to produce a synaptonemal complex [54]. Thus it is possible that these proteins play a role in chromosomal missegregation and crossover events in cancer.

## 7. Kinetochore/Spindle Assembly: A Different Orientation

The kinetochore is a proteinaceous structure which forms on centromeres and serves as a spindle fiber attachment site used to pull chromosomes apart and to opposite poles of the nucleus during cell division. Every chromatid has its own kinetochore, thus after the duplication of genetic material each chromosome contains two kinetochores, one on each chromatid. During mitosis, the two kinetochores on each chromosome face in opposite directions so that spindle fibers from each pole attach to separate chromatids. This is known as amphitelic attachment. The kinetochore works as a sensing mechanism, ensuring that the chromosomes are appropriately amphitelically attached. It does this by detecting the tension produced from the counteracting pull from each spindle into the bidirectionally oriented sister kinetochores. Only when this tension is sensed does the cell proceed through the spindle assembly checkpoint (SAC) and begin anaphase.

This process is more complicated in meiosis, as the cell must undergo two subsequent rounds of chromosome segregation without intervening DNA duplication. This is accomplished by a different orientation of the kinetochores in meiosis I through the use of REC8, monopolin proteins, and Aurora B Kinase [58, 65]. In meiosis I, sister kinetochores are oriented on the same side of the chromosome (monooriented) and attach to spindle fibers in a syntelic fashion to ensure that sister chromatids are pulled towards the same pole of the dividing cell. The tension necessary to proceed through the checkpoint is created by the crossovers between homologous chromosomes, which are pulled in opposite directions. Resolution of the chiasma allows for separation of the joined homologous pairs in anaphase I. Meiosis II then proceeds similarly to mitosis. 

Many of the details on kinetochore orientation and rotation in meiosis II have yet to be determined. REC8 is critical for mono-orientation [55, 58]. Condensins, proteins which play a major role in chromosome condensation and DSB repair during prophase I, are also known to play a role [66]. Brito et al. showed that in the absence of condensins, a portion of kinetochores biorient during meiosis I [66]. A third group of proteins called monopolins also play a key role [67–69]. Monopolins are meiosis specific proteins [69]. Through their interaction with Aurora B Kinase, monopolins help ensure that homologs are pulled towards opposite poles of the cell [67]. Although REC8 plays a clear role in maintaining monooriented sister kinetochores, monopolins alone are able to hold together sister kinetochores independently [67, 69].

Another critical part of this process is the spindle apparatus. In the female oocyte, the centrosome is destroyed before meiosis and the cell undergoes an acentrosomal spindle assembly [70, 71]. The spindle network is instead formed through the action of over 80 self-organized microtubule organized centers (MTOCs) that develop from the cytoplasmic microtubule complex and eventually aggregate into a bipolar network [70]. Very little is understood about acentrosomal spindle assembly and the differences between the mitotic and meiotic spindle, and further studies are needed.

During meiosis I, mechanisms exist to allow the unique process of homolog separation. Studies have shown that some SAC proteins specifically interact with REC8 and Shugoshin (SGO1 and 2) [56]. BUB1 is one of the SAC proteins shown to be specifically involved with this process. BUB1 is required for the localization of the meiosis cohesion regulators SGO1 and SGO2 to protect REC8 during meiosis I [72]. For this reason, BUB1 is thought to be essential for establishing proper kinetochore function [56, 73, 74]. BUB1 mutation results in chromosome fragmentation and missegregation in Drosophila [56, 73] and female specific germ cell aneuploidy in mice [56, 75].

BUB1 has been noted to be abnormally expressed in several cancers including gastric, colon, esophageal, breast, and melanoma [76–79]. The aberrant expression of BUB1 seems to have an especially strong correlation with melanoma [80]. Lewis et al. used qtPCR to identify molecular expression patterns in melanoma, benign nevi, and lymph nodes [80]. Of the 20 melanoma-related genes tested, BUB1 was one of the three genes found to have the highest discriminatory potential for distinguishing melanoma, benign nevi, and lymph nodes [80]. 

There is a delicate interplay between kinetochores, the spindle apparatus, and the SAC which, when not functioning properly, would be expected to result in chromosomal segregation abnormalities. 

## 8. Conclusion and Future Directions

Melanomas are highly genomically unstable tumors and are known to express germ cell proteins. It is possible that these two phenomena are related due to the collision of meiotic germ cell pathways with the normal mitotic cell cycle pathways (meiomitosis). As discussed, these pathways could impact genomic instability at many levels including double stranded DNA breaks, crossover events, chromosomal cohesion, spindle defects, and direct introduction of point mutations (Table 3). Chromosomal instability is a hallmark of human cancer, and we hypothesize that the aberrant expression of meiosis proteins will prove to be a critical step in this process. Expression of these pathways may serve a diagnostic role in identifying tumor with particular aggressive behavior due to their capacity to continuously evolve. Further, these pathways may serve as therapeutic targets. Because meiosis proteins are largely limited to germ cells, targeted therapeutics could be designed to have very limited interactions with other cells in the body reducing potential adverse side effects. Meiomitosis is likely to play a critical role in cancer progression and we believe future research is needed to shed light on this promising field.

## Figures and Tables

**Table 1:** Summary of the major events in each stage of the meiotic and mitotic cell cycle.

Stage of meiotic division	Outcome	Stage of mitotic division	Outcome
S Phase I	Duplication of genetic material, DNA double strand breaks induced by SPO11	S Phase	Duplication of genetic material

Prophase I	Chromosome condensation, homologous recombination	Prophase	Chromosome condensation

Metaphase I	Tetrad alignment at metaphase plate, cohesin degraded from chromosome arms but remains at centromere, monooriented sister kinetochores	Metaphase	Chromosome alignment at metaphase plate, cohesin degraded from centromere, bioriented sister kinetochores

Anaphase I	Homologous chromosomes separate to opposite sides of dividing cell, sister chromatids remain attached	Anaphase	Sister chromatids separate to opposite sides of dividing cell

Telophase I	Chromatid decondensation (sometimes just partial decondensation)	Telophase	Chromatid decondensation, two daughter cells are diploid

Prophase II	No chromosome duplication, chromosome recondensation

Metaphase II	Sister chromatids align at metaphase plate, cohesin degraded from centromere, bi-oriented sister kinetochores

Anaphase II	Sister chromatids separate to opposite sides of dividing cell

Telophase II	Chromosome decondensation, four daughter cells are haploid

**Table 2:** Summary of the differences between meiosis I, meiosis II, and mitosis.

	Meiosis I	Meiosis II	Mitosis
Reductional division	Yes	No	No
Equational division	No	Yes	Yes
Daughter Cells Genetically Identical	No	No	Yes
Double strand breaks introduced by SPO11	Yes	No	No
Pol *β* required for meiotic recombination and chromosome synapsis	Yes	No	No
Homologous chromosome recombination and segregation	Yes	No	No
Degradation of cohesion along chromosome arms	Yes	Not present	Not present
Degradation of cohesion at centromeres	No	Yes—during anaphase II	Yes—during anaphase
SGO2 protection of REC8 located at pericentromeric regions	Yes	No	No

**Table 3:** The Potential ramifications of the aberrant expression of different meiotic proteins.

Meiotic Pathway	Potential ramification in mitosis	Potential Meiotic Proteins involved
DS DNA strand breaks	Insertions, deletions, translocations	SPO11
Error prone polymerase	Point mutations	POLB
Failure of cohesin ring digestion	Tetraplody, polyploidy	REC8/SGO2
Unresolved chismata	Anaphase bridging	REC8
Failure of kinetochore separation	Chromosomal missegregation	REC8/SGO2
Misalignment of kinetochores	Chromosomal missegregation	REC8/monopolins
